# An education in tolerance: the 2025 Nobel Prize in Physiology or Medicine

**DOI:** 10.1242/dmm.052725

**Published:** 2025-11-19

**Authors:** Adrian Liston

**Affiliations:** Department of Pathology, University of Cambridge, Cambridge CB2 1QP, UK

## Abstract

The 2025 Nobel Prize in Physiology or Medicine has been awarded to Mary Brunkow, Fred Ramsdell and Shimon Sakaguchi “for their discoveries concerning peripheral immune tolerance”. This award celebrates research into the mechanisms by which the adaptive immune system learns to tolerate self-antigens, preventing *horror autotoxicus*, or autoimmune disease. The identification of the regulatory T cell, a type of white blood cell with the capacity to impose self-tolerance on immune responses in the peripheral blood and tissues, required a combination of innovative immunology and genetics. The decades-long task of characterising this cell that can shut down autoimmunity finally intersected with the genetic mapping of the gene *FOXP3*, which underlies a rare autoimmune condition in humans and mice. This fusion of mouse models and patient-based genetic analysis set off an explosion of research into immune regulation, which is still redefining our knowledge of biology and medicine.

## Teaser

**Summary:** The 2025 Nobel Prize in Physiology or Medicine has been awarded to Mary Brunkow, Fred Ramsdell and Shimon Sakaguchi to honour the research leading up to the discovery of the regulatory T cell.

This year, the Nobel Prize in Physiology or Medicine was awarded to Mary E. Brunkow, Fred Ramsdell and Shimon Sakaguchi “for their discoveries concerning peripheral immune tolerance”. This award honours the research leading up to the discovery of a rather unique type of immune cell, the regulatory T cell. This white blood cell has a profoundly different role in the immune system from other immunological cell types. Whereas for most immune cells, their primary function lies in their ability to initiate or propagate an inflammatory reaction that can combat infections, the regulatory T cell has the reverse function. Regulatory T cells can suppress the activation of other immune cells and initiate healing and rejuvenation. In effect, the regulatory T cell acts as the brakes on the immune system, shutting down inflammation before it becomes too damaging, and instructing other immune cell types to calm down and allow repair processes to be initiated. In the absence of regulatory T cells, the mammalian immune system becomes uncontrollable, driving a fatal multi-tissue self-destruction sequence.

**Fig. 1.**
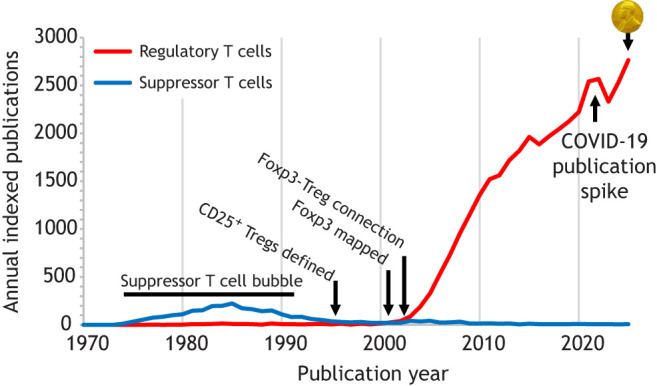
**Publication timeline for work on regulatory T cells.** Annual publications based on PubMed indexation. Regulatory T cell publications include mentions of either ‘regulatory T cell’ or ‘Treg’. 2025 data are extrapolated from publication data in January to October. Key dates are indicated on the graph.

Despite the fundamental importance of the regulatory T cell to the human immune system, the pathway to discovery of this cell type was a rocky one. The concept of a T cell subset with unique tolerogenic properties had been long considered. Proposed ‘suppressor T cells’ were actively researched in the 1970s and 1980s (Fig. 1), with a proposed gene, the I-J locus, being at the centre of their suppressive capacity (Murphy et al., 1976). The genetic revolution, however, was unkind to the suppressor T cell, and the I-J locus was demonstrated to not exist in 1983 (Kronenberg et al., 1983). Around this time, solid experimental evidence for a long theorised, alternative tolerance mechanism was coming out of the teams led by Harald von Boehmer, Pippa Marrack and John Kappler (Kappler et al., 1987; Kisielow et al., 1988). This established ‘central tolerance’ as a bona fide T cell tolerance mechanism, which relied on autoreactive T cells being completely removed during differentiation in the thymus. With suppressor T cells in disgrace, and central tolerance experimentally validated, the support for the concept of an additional immune tolerance mechanism acting in the periphery faded. Suppressor T cells became a cautionary tale of chasing non-reproducible phenotypes with excessive optimism, and research on the topic dwindled (Fig. 1).

Despite this ignominy, it is important to note that many of the findings during this period have stood the test of time and did not rely on the mythical I-J locus. Embryologist Nicole Le Douarin demonstrated that transplantation of a thymic lobe prevented graft rejection in her quail-chicken chimeric experiments (Ohki et al., 1987, 1988). As the quail-chicken has multiple anatomically discrete thymic lobes, this result could not be accounted for by central tolerance (deletion of quail-reactive T cells) and necessitated a suppressive T cell to be educated in the thymus to stop transplant rejection in the periphery. At the same time, the teams of Shimon Sakaguchi, Don Mason and Fiona Powrie were tracking down subsets of T cells with suppressive properties from rodent models. Their work sought to purify T cells that had the capacity to shut down autoimmunity and wasting in rat and mouse models of immune disease (Powrie and Mason, 1990; Sakaguchi et al., 1982). These farsighted experiments kept the field alive at a time when research into the topic had all but ceased (Fig. 1). From our current perspective, it is difficult to understand just how profoundly unpopular the topic was, and the degree to which these pioneering groups had to swim against the tide in order to continue their research.

Sakaguchi and Powrie, in particular, did persevere, and progressively recreated the field of suppressor T cells, now rebranded as regulatory T cells, and built on a more solid experimental foundation. A major breakthrough in the field came in 1995, when Sakaguchi's team identified CD25, the alpha chain of the interleukin 2 receptor, as a protein that largely distinguished regulatory T cells from the conventional, inflammatory, T cells (Sakaguchi et al., 1995). This marker was much more amenable to the type of fractionation experiments required at the time, allowing regulatory T cells to be purified and transferred in multiple disease models. The work attracted new groups to the field and even allowed the discovery of key effector molecules, including CTLA4 (Read et al., 2000; Takahashi et al., 2000). The field had become rehabilitated, and although publication numbers were at a near-low, the turning point had been reached (Fig. 1).

Whereas Sakaguchi's Nobel Prize was rooted in immunology, the recognition of Brunkow and Ramsdell emerged from genetics research. Independent of the work on suppressor T cells, a seemingly unrelated immune phenomenon was being researched: a rare genetic disorder called ‘immune dysregulation, polyendocrinopathy, enteropathy, X-linked syndrome’ (IPEX). By coincidence, a mouse strain, named *Scurfy* mice, with similar disease and inheritance pattern, had been created following radiation exposure in the Manhattan Project. In 2001, Brunkow and Ramsdell published a study that mapped the *Scurfy* mutation down to the gene *Foxp3*, formally demonstrating that replacing the mutated gene prevented disease development (Brunkow et al., 2001). The work was rapidly translated to IPEX, demonstrating that mutations in the human homologue *FOXP3* were the drivers of immunological disease in patients (Bennett et al., 2001; Wildin et al., 2001).

At this point, the function of FOXP3 was still rather mysterious – although it was clear that mutations in *FOXP3* resulted in immunological disease, the mechanism remained unknown, in humans and mice. The rescue of disease by transplantation of healthy bone marrow demonstrated an immune-intrinsic function of the gene, encouraging three groups, led by Sakaguchi, Ramsdell and Sasha Rudensky, to test a potential link between Foxp3 and regulatory T cells. In 2003, all three groups demonstrated this connection, identifying Foxp3 as the master transcription factor that enabled conventional T cells to be converted into regulatory T cells (Fontenot et al., 2003; Hori et al., 2003; Khattri et al., 2003). The induction of this transcription factor in self-reactive T cells during thymic differentiation rewired the T cell in a way that inverts function, causing self-antigen exposure recognition to initiate the expression of suppressive, rather than inflammatory, mediators.

The molecular link between Foxp3 and regulatory T cells was the key to unlocking research via mouse genetic tools. Within only a few years, a flurry of genetic tools had been created, including floxed alleles, promoter element knockout alleles, expression-tracing alleles (GFP, YFP, RFP, Thy1.1, luciferase), Cre alleles (constitutive and inducible), deletion-inducing alleles (DTR, iCaspase9) and more (Jeremiah and Liston, 2011). These mouse strains, made and widely distributed by multiple groups, but in particular those led by Rudensky, Sparwasser and Flavell, transformed the field. Suddenly, regulatory T cells could be readily quantified and visualised, transferred and depleted. Candidate effector molecules could be depleted from regulatory T cells, and the molecular underpinning of their induction, homeostasis and effector molecules could be dissected. This initiated a surge in research into regulatory T cells that has continued to escalate unablated for more than two decades (Fig. 1).

The impact of this research on our mechanistic understanding of disease has been profound. Deficits in regulatory T cells, either numerical or functional, have been associated with multiple autoimmune or inflammatory diseases, while recruitment of regulatory T cells is now known to protect tumours from immune rejection (Sakaguchi et al., 2020). We have an in-depth understanding of the various pathways that can convert a conventional T cell to the regulatory fate, and a growing understanding of the molecular mechanisms of immune suppression (Josefowicz et al., 2012). These advances have been put into clinical practice – low-dose interleukin 2 and regulatory T cell-based cell therapies have both been used effectively to prevent conditions, such as graft-versus-host disease (Klatzmann and Abbas, 2015; Raffin et al., 2020), and CTLA4 has been exploited in both an agonistic and antagonistic format to retune the immune system during inflammation (Bluestone et al., 2006) or cancer (Sharma et al., 2023), respectively. At the same time, there is still much to learn about these enigmatic cells. In particular, little is known about which antigens are recognised by regulatory T cells, other than them generally being self-antigens. This fundamental question of immune regulation will likely have great influence on the design of cell therapies, as regulatory T cells are engineered to improve homeostasis, localisation and targeting of immunosuppressive effect. Likewise, although the key effector mechanisms for immunosuppression have been well studied, very little is known about the molecular systems that regulatory T cells use to enhance tissue repair mechanisms – another fundamental knowledge gap that could potentially drive therapeutic development if filled.

The Nobel Prize was a recognition of the inspiring science that underlay the discovery of regulatory T cells, owing to the clinical impact these cells already have, and are likely to have through further discovery and therapeutic development. Although the Prize was limited to Brunkow, Ramsdell and Sakaguchi, the Nobel citation celebrates the many teams of researchers who transformed this field. The selection of both geneticists and immunologists to headline with the award also recognises the powerful synergy of combining human genetics with mouse models of cellular immunity. Perhaps the most important recognition, however, lies in the endorsement of slow science, of persevering against strong headwinds and rebuilding a fallen hypothesis with care and creativity.
